# Niclosamide Treatment Suppressed Metastatic, Apoptotic, and Proliferative Characteristics of MDA-MB-231 Cancer Stem Cells

**DOI:** 10.1021/acsomega.5c02545

**Published:** 2025-05-28

**Authors:** Özlem Altundag-Erdogan, Betül Çelebi-Saltik

**Affiliations:** † Hacettepe University, Graduate School of Health Sciences Department of Stem Cell Sciences, Sihhiye, Ankara, TR 06100, Turkey; ‡ Hacettepe University, Center for Stem Cell Research and Development, Sihhiye, Ankara, TR 06100, Turkey

## Abstract

This study evaluated the efficacy of niclosamide after the invasion of aggressive TNBC breast CSCs into a 3D bone-mimicking model. Initially, the optional dose required for triggering apoptosis was determined for MDA-MB-231 CSCs (CD44+ and CD24−). Our findings revealed that approximately 50% of the cells showed apoptotic properties, as assessed with Annexin V/7AAD assay and WST-1 at IC_50_ = 100 μM (6 h). Additionally, this treatment suppressed p-STAT3 protein levels and increased Bax levels (*p* < 0.05), as determined by Western Blot. The expression of genes associated with metastasis and cell migration (*CXCR4, MMP2, MMP9*), drug resistance (*ABCG1, ABCG2*), stemness (*OCT4, NANOG*) and cell cycle and proliferation (*CYCLIN D1*) was found to be significantly suppressed (*p* < 0.05). Therefore, after validating the efficacy of the 100 μM dose on CSCs, cell cycle, ELISA, Western Blot, and RT-qPCR analyses were conducted in the 3D model. It was found that the cells were arrested in the G0–G1 phase (*p* < 0.05). 100 μM Niclosamide suppressed the levels of EMT markers, Vimentin (*p* > 0.05) and ZEB1 (*p* < 0.05). Additionally, RT-qPCR results indicated a significant downregulation of *CXCR4, ABCG1, ABCG2, MMP2, OCT4, CCND1, AXIN2, and LGR5* gene expressions following niclosamide treatment in both CD133+ and CD133– groups (*p* < 0.05). The increase in the Bax protein, a key player in apoptosis induction, along with the decrease in the anti-apoptotic protein Bcl-2, suggests the activation of cell death mechanisms. Notably, its targeted impact on the CD44+/CD24– population suggests that niclosamide could enhance the sensitivity of CSCs to treatment, thereby preventing tumor recurrence.

## Graphical abstract

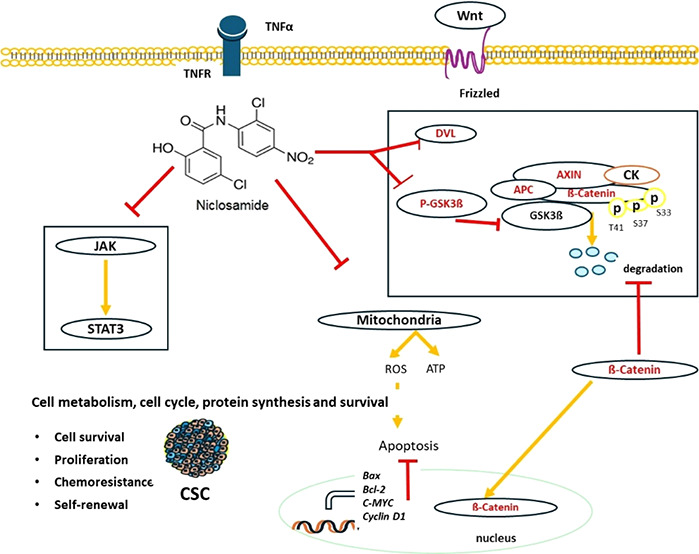

## Introduction

Triple-negative breast cancer (TNBC) lacks receptors for estrogen, progesterone, and HER2, making it unresponsive to common hormonal therapies.^1^ Thus, TNBC is associated with poor prognoses due to its aggressive nature, limited targeted therapy options, high therapeutic resistance, and propensity for metastasis, which complicates treatment responses. Given that TNBC contains a higher proportion of cancer stem cells (CSCs), targeting these cells is crucial to improving therapeutic outcomes and preventing relapse.^2,3^ Breast CSCs, characterized by the CD44+ and CD24–/low cell surface protein expression signature, play a critical role in tumor heterogeneity, therapeutic resistance, and disease recurrence, posing significant challenges in breast cancer treatment. Their dormancy in the G0/G1 phase, which renders them less sensitive to therapies targeting rapidly dividing cells, allows them to evade treatments such as chemotherapy that focus on proliferative tumor cells.^4^ Besides, the tumor microenvironment plays a critical and indispensable role in homing, survival, proliferation, and development of drug resistance in cancer cells. In bone marrow niche, multiple cell types provide shelter to protect cells from chemotherapy treatment. These include osteoblasts, MSCs, tumor-associated macrophages (TAMs), and ECM structures and proteins that can directly affect the response and apoptosis pathways of cancer cells and allow them to remain dormant. Therefore, targeting these CSCs in the 3D model is a meaningful strategy.^5,6^

A recent study hypothesized that the suppression of canonical Wnt/β-catenin and STAT3 activity by niclosamide would have cytotoxic potential alone and would sensitize BLBC stem cells to treatment with TRA-8.^7^ It has been reported that the combination of niclosamide and paclitaxel is more effective than paclitaxel alone. Niclosamide inhibits the proliferation of paclitaxel-resistant esophageal cancer cells and triggers and initiates apoptosis, increasing the in vivo efficacy of paclitaxel. Niclosamide significantly inhibited the growth of paclitaxel-resistant esophageal cancer in mice by targeting the Wnt/β-catenin pathway and did not cause toxicity.^4^ In addition, niclosamide can increase the sensitivity of cervical cancer cells to paclitaxel by inhibiting the mTOR pathway.^8^ This suggests that paclitaxel or doxorubicin and niclosamide can be used in chemotherapy for TNBC by inhibiting cell proliferation and migration and reducing the expression of Ki67 and CD44 to induce apoptosis.^9^ Niclosamide, has been repurposed for cancer treatment due to its ability to disrupt multiple oncogenic signaling networks, such as Wnt/β-catenin, NF-κB and STAT3, all of which has fundamental roles in the survival, proliferation, and invasiveness and metastasis of tumors.^10^ Several preclinical studies support that STAT3 is persistently activated in CSC-enriched populations of TNBC, while blocking or reducing STAT3 signaling suppresses CSC burden, EMT, tumor growth, and metastasis.^11,12^ After being transported to the nucleus, activated p-STAT3 upregulates the expression of key genes, including *CCND1* (cyclin D1), *BCL* *2*, *BIRC5* (survivin), and *SOX2*, which are responsible for cell growth, differentiation, EMT, and apoptosis.^13–16^

Although niclosamide has shown promise in inhibiting survival pathways in various cancer types, its specific impact on cancer cells remains incompletely characterized.^17,18^ In our previous publication, we used a 3D model to investigate the therapeutic efficacy of the mTOR inhibitor temsirolimus on CSCs. To mimic bone tissue, osteogenesis was induced by MSCs for 21 days, followed by 1 week of HUVEC culture, and then aggressive CSC invasion was performed to mimic the tumor microenvironment. In this study, we targeted CSCs with niclosamide in our 3D model. Then, we evaluated the effect of the drug with flow cytometry (Annexin-V/7AAD, cell cycle), Western blot (apoptotic, STAT-3, Wnt, EMT proteins), and RT-qPCR (drug resistance, stemness, cell migration, invasion, and cell cycle genes) experiments.

## Results

### Inhibition of STAT3 and Induction of Apoptosis in Cancer Stem Cells Following Niclosamide Treatment

The CSCs with surface marker profiles of CD44+ (99.7%) and CD24–/low (0.2% positive) were morphologically characterized as displaying a spheroid form under suspension culture conditions (Figure 1a). As part of our preliminary studies, we treated the isolated CSCs with niclosamide to determine the effective dose, which was determined by assessing cell viability using the Annexin V/7AAD assay. Our findings revealed that after applying 100 μM niclosamide, the percentage of viable cells was 53.4 ± 1.2%. Consequently, the IC_50_ value for the 6 h of treatment was determined to be this dose (Figure 1b). Subsequently, WST-1 and Western blot data at this dose were presented. According to our WST-1 results, the absorbance decreased to 50% of the baseline value (*p* < 0.05, Figure 1c).

**Figure 1:**
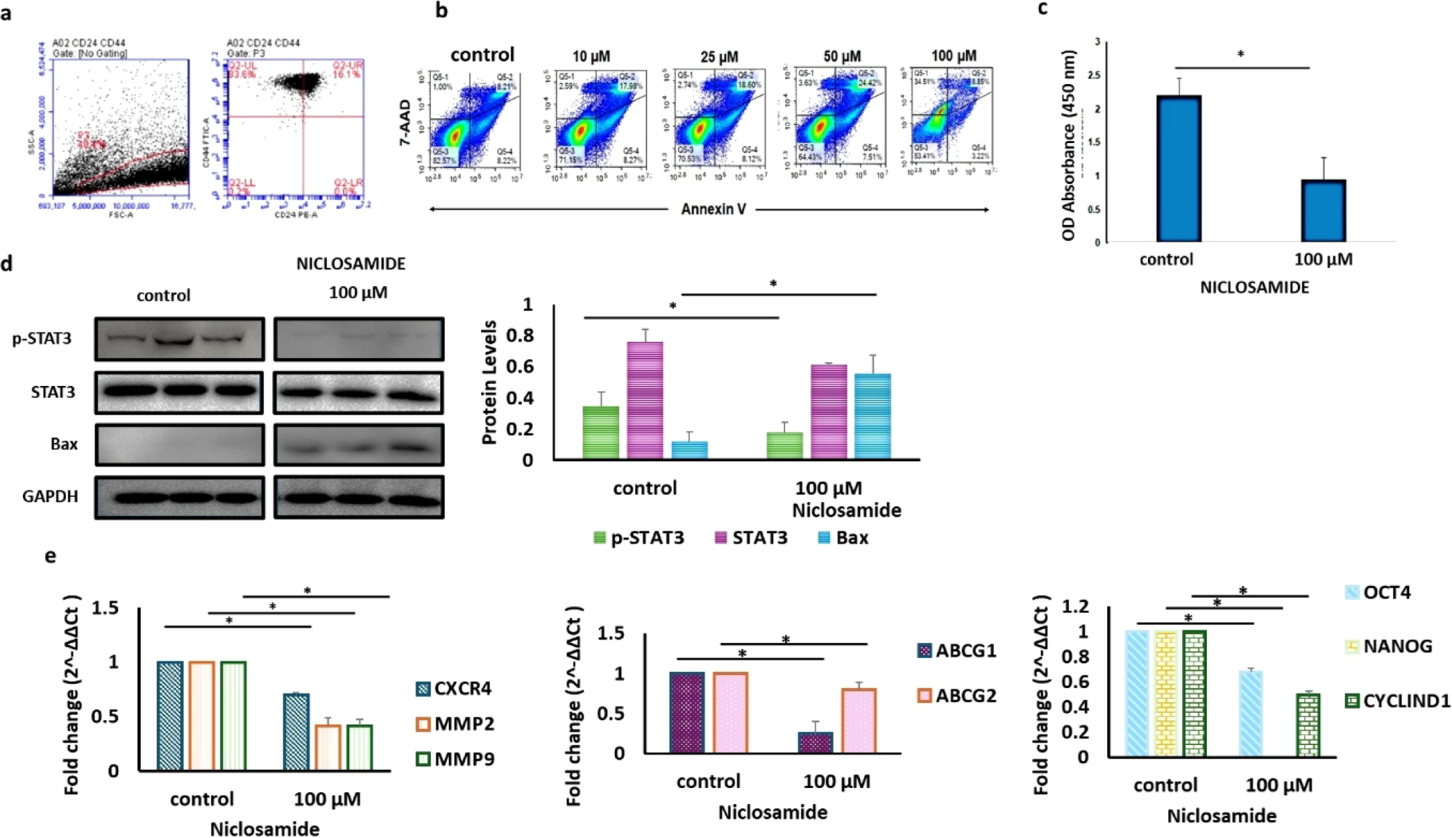
Evaluation of cell survival, apoptosis, and stemness. a) Characterization of CSCs (CD44+/CD24–) by flow cytometry. b) Annexin V-FITC/7AAD assay for the 0–100 μM dose interval for 6 h of treatment. c) WST-1 assay results for the control and 100 μM treatment. d) Protein levels of CSCs after control and 100 μM niclosamide treatment (mean ± std, *n* = 3, *: *p* < 0.05). e) The effect of niclosamide on gene expression in CSCs. Metastasis and cell migration (left), drug resistance (middle), and stemness- and proliferation-related genes (right). p-STAT3: phosphorylated signal transducer and activator of transcription 3, STAT3: signal transducer and activator of transcription 3, Bax: Bcl-2 associated X protein, and GAPDH: glyceraldehyde-3-phosphate dehydrogenase.*ABCG1*: ATP binding cassette subfamily G member 1, *ABCG2*: ATP Binding cassette subfamily G member 2, *CXCR4*: C-X-C chemokine receptor type 4, *MMP2* matrix metallopeptidase 2, *MMP9*: matrix metallopeptidase 9, *OCT4:* octamer-binding transcription factor 4, and *NANOG* nanog homeobox.

The effect of 100 μM niclosamide as a STAT3 inhibitor was further evaluated using Western blot analysis, and cells were treated for 6 h. Figure 1c presents representative protein bands along with the corresponding histogram data, illustrating the changes in p-STAT3, STAT3, and Bax protein levels (Figure 1d). Treatment with niclosamide significantly reduced p-STAT3 levels (*p* < 0.05) and simultaneously increased the levels of the proapoptotic protein Bax (*p* < 0.05). Based on these findings, 100 μM niclosamide was considered an effective dose for testing in the 3D model and was applied in subsequent experiments. Besides, following 100 μM niclosamide treatment, RT-qPCR analysis revealed a decrease in the expression of the following genes: *CXCR4* (1.5-fold*), MMP2* (1.3-fold), *MMP9* (2.9-fold), *OCT4* (1.5-fold), *CYCLIN D1* (2-fold), *ABCG1* (3.7-fold), and *ABCG2* (1.3-fold) (*p* < 0.05, Figure 1d) in CSCs.

### Invasion of Cancer Stem Cells in the Three-Dimensional Model

The outer 3D-printed scaffold (PLA) was characterized in our earlier study (Figure 2a) and was integrated with a PU vascular system fabricated via the electrospinning method, which was inserted through the center of the structure to facilitate drug release. The internal space was filled with a COL/γ-PGA/Na_2_SiO_3_-based composite. Mesenchymal stem cells (MSCs) were seeded for 21 days to induce osteogenic differentiation, while vascularization was established through coculture with HUVECs. This 3D model has been fully characterized in our previous work.^19^ In the current experimental setup, isolated CSCs were first introduced into the system (Figure 2b), and subsequently, CSCs within the 3D structure were targeted with niclosamide (IC_50_ = 100 μM, 6 h).

**Figure 2:**
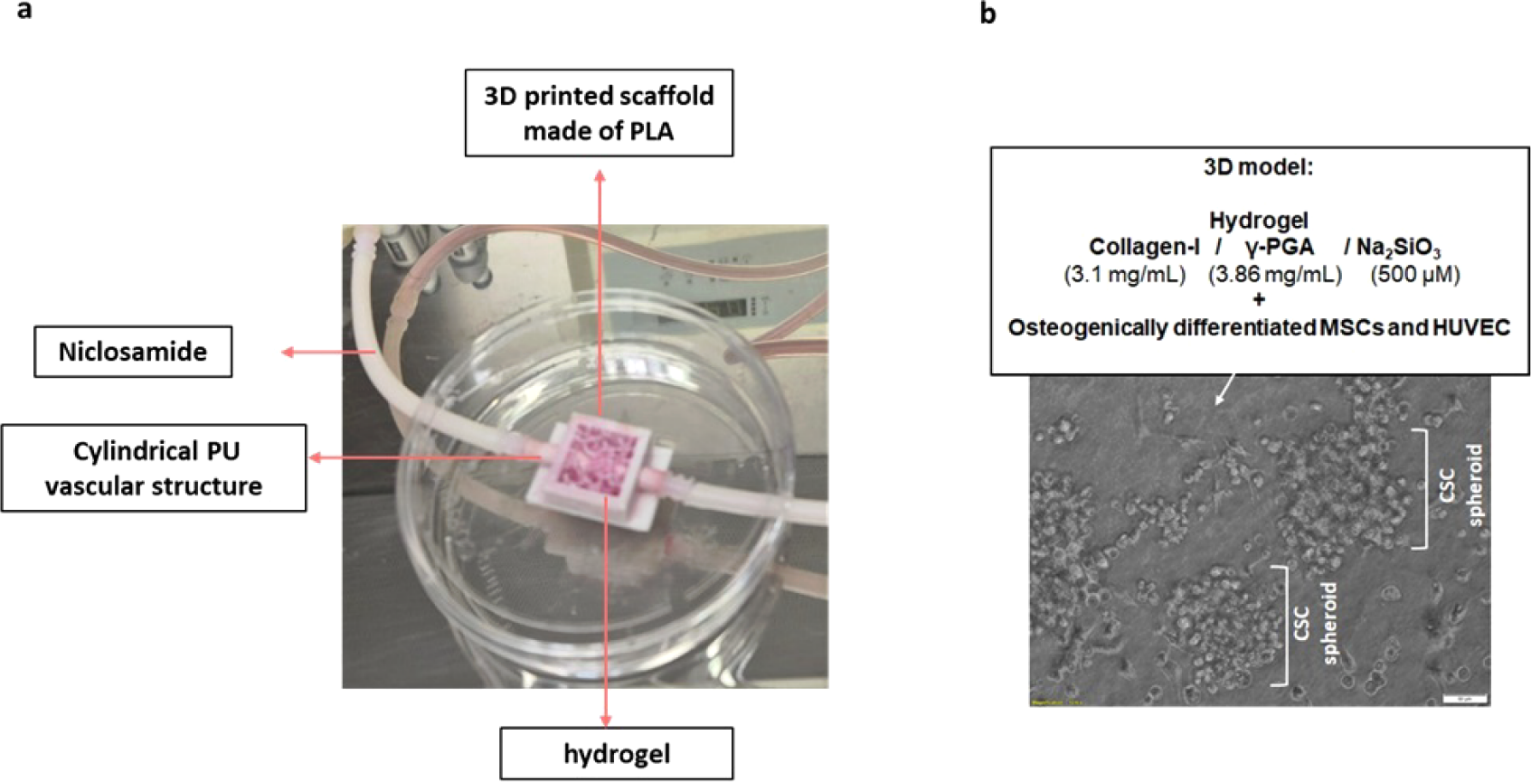
Cancer stem cells in the 3D model. a) 3D model of the system. b) Imaging for assessment of CSC spheroids on the material surface. CSC spheroids invading the structure are indicated with white corner brackets. Diameter of spheroids = 131.5 ± 5.4 μm (mean ± std, *n* = 3). Scale bar= 50 μm.

### Niclosamide Treatment Induced G0/G1 Arrest in Cancer Stem Cells

Cell cycle analysis was performed before and after niclosamide treatment by flow cytometry to determine the number of cells in the G0, G1, S, and G2/M phases (Figure 3). In the control group, the frequency of cells in G0/G1 (45.2 ± 1.1), S (5.9 ± 1.3), and G2/M (18.4 ± 2.1) was observed, while following drug treatment, the frequency changed to G0/G1 (61.4 ± 3.2), S (5.8 ± 1.1), and G2/M (20.64 ± 1.8).

**Figure 3:**
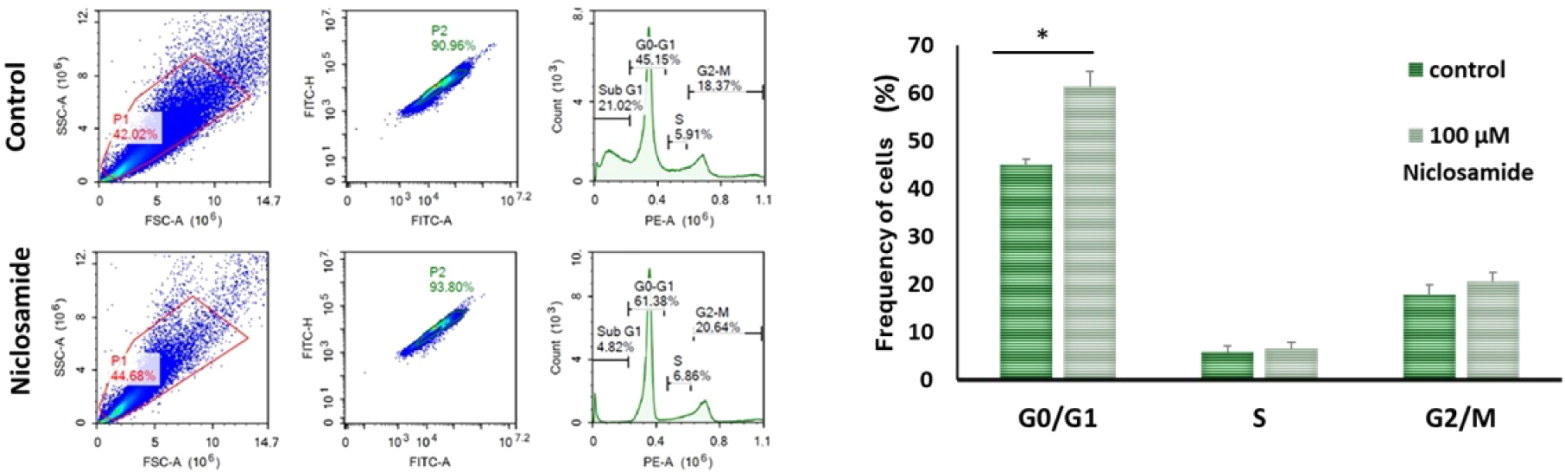
Distribution of cells in G0/G1, S, and G2/M phases before and after niclosamide treatment, represented by representative images (left) and histogram data (right). Control: cells cultured in a drug-free medium. 100 μM: cells treated with 100 μM niclosamide for 6 h (mean ± std, *n* = 3, *: *p* < 0.05).

### Effects of Niclosamide on Cancer Stem Cell Protein Levels in 3D Culture

In this study, niclosamide, a known STAT3 and Wnt pathway inhibitor, was employed to evaluate the drug response of CSCs within the bone-mimetic 3D model. In the 3D culture system treated with 100 μM niclosamide, a decreasing trend was observed in the protein levels of vimentin (1.3-fold) and EpCAM (1.4-fold), while no significant variation was noted in the stemness-associated ALDH1A1 protein levels. However, a reduction in the levels of C-Myc (1.3-fold), Bcl-2 (1.6-fold), ZEB1 (2.5-fold), and p-GSK3ß (2.2-fold) was observed (*p* < 0.05), whereas the Bax protein levels increased significantly (5.7-fold, *p* < 0.05, Figure 4).

**Figure 4:**
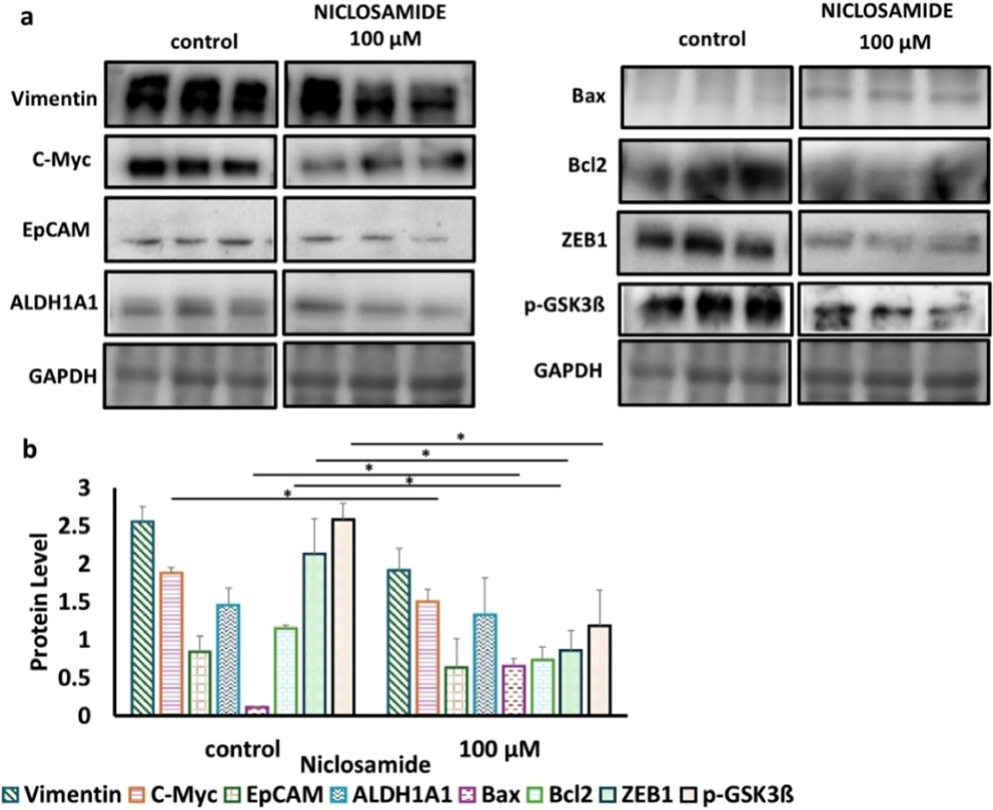
Evaluation of protein levels before and after niclosamide treatment. a) Representative images and b) histogram of the changes in Wnt/β-catenin and metastasis-related protein levels. Control. (mean ± std, *n* = 3, *: *p* < 0.05). C-Myc: cellular myelocytomatosis proto-oncogene, EpCAM: epithelial cell adhesion molecule, ALDH1A1: aldehyde dehydrogenase 1 family member A1, GAPDH: glyceraldehyde-3-phosphate dehydrogenase, Bax: Bcl-2-associated X protein, Bcl-2: B-cell lymphoma 2, ZEB1: zinc finger E-box binding homeobox 1, and p-GSK3ß: phosphorylated glycogen synthase kinase 3 beta.

The evaluation of metastasis-associated cytokines, chemokines, and growth factors released into the supernatant in the 3D environment before and after niclosamide treatment was performed by measuring the levels of IL-8, IL-6, IL-1ß, IL-18, and VEGF (Table 1). According to our ELISA results, significant increases in cytokine levels were observed following niclosamide treatment.

**Table 1:** Cytokine Secretion of Cells before and after Niclosamide Treatment (*n* = 3, Mean ± Std, *: *p* < 0.05)

Cytokines	Control	Niclosamide (100 μM)
IL-8	463.36 ± 42.22 ng/L	508.77 ± 55.08 ng/L
IL-18	83.80 ± 6.96 ng/L	91.26 ± 1.16 ng/L
IL-1ß	4559.40 ± 2.09 pg/mL*	6399.00 ± 0.09 pg/mL*
IL-6	241.77 ± 7.27 ng/L*	422.85 ± 28.79 ng/L*
VEGFA	264.09 ± 20.24 ng/L*	425.57 ± 24.28 ng/L*

The evaluation of metastasis-related cytokine, chemokine, and growth factor secretion in the 3D environment before and after niclosamide treatment was performed by assessing IL-8, IL-6, IL-1ß, IL-18, and VEGF levels in the supernatant (Table 2). According to our ELISA results, significant increases in cytokine levels were observed following niclosamide treatment compared with the control group.

**Table 2:** Primer Sequences Used for Gene Expression Analysis in RT-qPCR

	Forward	Reverse
**CXCR4**	CTCCTCTTTGTCATCACGCTTCC	GGATGAGGACACTGCTGTAGAG
**ABCG1**	GAGGGATTTGGGTCTGAACTGC	TCTCACCAGCCGACTGTTCTGA
**ABCG2**	GTTCTCAGCAGCTCTTCGGCTT	TCCTCCAGACACACCACGGATA
**MMP2**	AGCGAGTGGATGCCGCCTTTAA	CATTCCAGGCATCTGCGATGAG
**MMP9**	GCCACTACTGTGCCTTTGAGTC	CCCTCAGAGAATCGCCAGTACT
**OCT4 (POU5F1)**	CCTGAAGCAGAAGAGGATCACC	AAAGCGGCAGATGGTCGTTTGG
**NANOG**	CTCCAACATCCTGAACCTCAGC	CGTCACACCATTGCTATTCTTCG
**CYCLIN D1**	TGAACTACCTGGACCGCT	GCCTCTGGCATTTTGGAG
**AXIN2**	CGACAGTGAGATATCCAGTGATG	TCTCTGGAGCTGTTTCTTACTG
**LGR5**	GGAATGTTTCAGGCTCAAGATG	TCAAGCAGGTGTTCACAGG
**GAPDH**	GTCTCCTCTGACTTCAACAGCG	ACCACCCTGTTGCTGTAGCCAA

### Differential Inhibitory Effects of Niclosamide on Cell Migration, Invasion, Cell Cycle, and Stemness Genes between CD133+ and CD133– Groups

The provided histogram data compare the fold changes (calculated as 2^–ΔΔCt^) in gene expression levels across control and 100 μM niclosamide treatment groups for various gene targets. The expression of *CXCR4* (57-fold), *MMP2* (2.8-fold), ABCG1 (2.7-fold), *OCT4* (2.2-fold), *NANOG* (6.3-fold), and *CYCLIN D1* (4.6-fold) was significantly downregulated (*p* < 0.05). *MMP9* expression was 1.9-fold higher in niclosamide-treated cells compared to the control (*p* < 0.05, Figure 5). However, *MMP9* expression was 1.9-fold higher than that of the control in the treatment group. *AXIN2* expression demonstrated a profound downregulation in the coculture conditions compared to the control group, with a fold decrease of approximately 5-fold (*p* < 0.05). In addition, *LGR5* expression was even more markedly suppressed, exhibiting an approximately 10-fold decrease relative to the control (*p* < 0.05).

**Figure 5:**
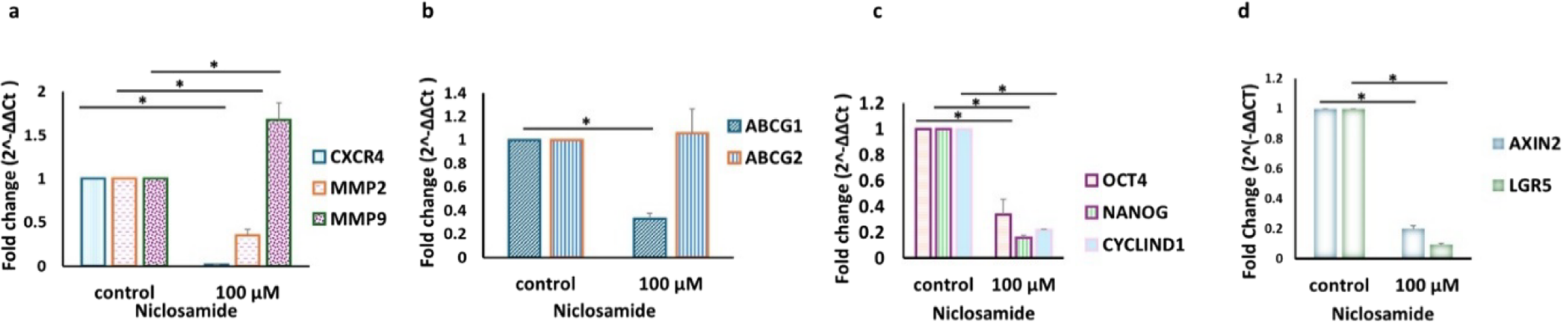
Detection of gene expression changes in the 3D model following niclosamide treatment. RT-qPCR results for a) cell migration and metastasis, b) drug resistance, c) stemness and cell proliferation and d) Wnt target genes. *ABCG1*: ATP binding cassette subfamily G member 1, *ABCG2*: ATP binding cassette subfamily G member 2, *CXCR4:* C-X-C chemokine receptor type 4, *MMP2* matrix metallopeptidase 2, *MMP9:* matrix metallopeptidase 9, *OCT4:* octamer-binding transcription factor 4, *NANOG*: nanog homeobox, *AXIN2* axis inhibition protein 2, and *LGR5* leucine-rich repeat-containing G-protein coupled receptor 5. Control: protein levels in cells cultured in a drug-free medium.100 μM: protein levels in cells treated with 100 μM niclosamide for 6 h (mean ± std, *n* = 3, *: *p* < 0.05).

In the CD133– cell group, after niclosamide treatment, downregulation of gene expression was observed for *ABCG1* (2.7-fold), *ABCG2* (2.2-fold), *OCT4* (1.3-fold), and *CYCLIN D1* (2.4-fold), and upregulation of *MMP9* expression (1.5-fold) was significant. Furthermore, although there was a trend toward a decrease in gene expression for CXCR4 (1.44-fold) and MMP2 (1.35-fold), these changes were not statistically significant (*p* > 0.05, Figure 6). Both *AXIN2* and *LGR5* expressions after niclosamide treatment resulted in marked downregulation, with the expression levels reduced to approximately a 5-fold decrease (*p* < 0.05).

**Figure 6:**
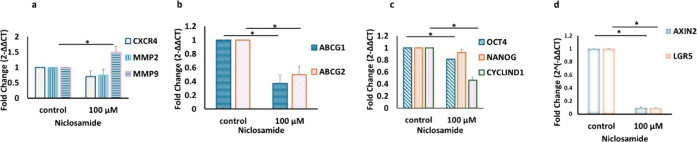
Changes in gene expression in CD133– coculture cells before and after niclosamide treatment. RT-qPCR results for a) cell migration and metastasis, b) drug resistance, c) stemness and cell proliferation, and d) Wnt target genes. *ABCG1*: ATP binding cassette subfamily G member 1, *ABCG2*: ATP binding cassette subfamily G member 2, *CXCR4:* C-X-C chemokine receptor type 4, *MMP2* matrix metallopeptidase 2, *MMP9:* matrix metallopeptidase 9, *OCT4:* octamer-binding transcription factor 4, *NANOG*: nanog homeobox, *AXIN2* axis inhibition protein 2, and *LGR5* leucine-rich repeat-containing G-protein coupled receptor 5. Control: protein levels in cells cultured in a drug-free medium.100 μM: protein levels in cells treated with 100 μM niclosamide for 6 h (mean ± std, *n* = 3, *: *p* < 0.05).

In the CD133+ cell group, niclosamide treatment (100 μM, 6 h) caused a decrease in the expression levels of *CXCR4* (5-fold), *MMP2* (5.3-fold), *ABCG1* (4.9-fold), *OCT4* (4-fold), *ABCG2* (5.2-fold), and *NANOG* (2-fold) (*p* > 0.05, Figure 7). Besides, the expression levels of *AXIN2* (3.3-fold) and *LGR5* (10-fold) were reduced following niclosamide treatment (*p* < 0.05).

**Figure 7:**
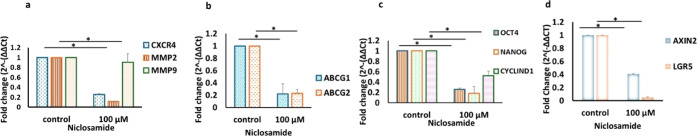
Changes in gene expression in CD133+ coculture cells before and after niclosamide treatment. RT-qPCR results for a) cell migration and metastasis, b) drug resistance, c) stemness and cell proliferation, and d) Wnt target genes. *ABCG1*: ATP binding cassette subfamily G member 1, *ABCG2*: ATP binding cassette subfamily G member 2, *CXCR4:* C-X-C chemokine receptor type 4, *MMP2* matrix metallopeptidase 2, *MMP9:* matrix metallopeptidase 9, *OCT4:* octamer-binding transcription factor 4, *NANOG*: nanog homeobox, *AXIN2* axis inhibition protein 2, and *LGR5* leucine-rich repeat-containing G-protein coupled receptor 5. Control: protein levels in cells cultured in a drug-free medium.100 μM: protein levels in cells treated with 100 μM niclosamide for 6 h (mean ± std, *n* = 3, *: *p* < 0.05).

## Discussion

While breast cancer remains a leading cause of cancer-related death, TNBC is the focus of studies due to the lack of targeted therapies.^20–23^ TNBC tumors typically have a high CSC-like population and tend to exhibit high drug resistance. Additionally, TNBC patients are relatively more likely to develop metastases following chemotherapy.^24,25^ STAT3 is predominantly activated through the phosphorylation of a tyrosine residue (Tyr705).

In our study, we employed a well-established in vitro model to enrich CSCs by culturing in serum-free media with growth factors on low-attachment plates, promoting the formation of spheroids rather than adherent individual cells.^26^ To further isolate CSCs, we performed CD133 selection, a marker linked to stemness, after targeting within the 3D model. In this study, our results showing the rationale for selecting the dose of IC_50_ = 100 μM (6 h) in isolated CSCs are shown in Figure 1. Annexin-V/7AAD staining detected 53.4 ± 1.2% cell viability after niclosamide treatment. We detected a significant decrease in absorbance after niclosamide treatment with WST-1 detection, indicating a decrease in metabolic activity to 50% of the initial level (*p* < 0.05, Figure 1b). This decrease in absorbance is proportional to the number of viable cells. In a study, treatment of colon cancer cells with niclosamide resulted in significant growth inhibition and apoptosis induction, and these effects were confirmed by the MTT and Annexin V-FITC assays.^27^ In another study, niclosamide-treated TNBC xenograft tumors exhibited reduced angiogenesis and tumor growth, as well as decreased Ki-67 expression and increased apoptosis. Additionally, distant metastases were suppressed in TNBC allotransplants derived from a CSC-enriched population. Niclosamide treatment in breast cancer has been shown to inhibit cell proliferation by modulating apoptosis-related proteins such as cleaved caspase-3 and Bcl-2, ultimately leading to cell death. It was reported that niclosamide treatment (30 μM, 48 h) was found to disrupt stemness pathways in MCF-7 and MDA-MB-231 CSCs, reduce spheroid formation, and induce apoptosis.^28^ Furthermore, a recent study on MDA-MB-231 breast cancer cells demonstrated that niclosamide concentrations ranging from 0 to 100 μM effectively inhibited wound healing, cell viability, spheroid formation, and stem cell-like properties at both the 8 h and 24 h treatment intervals. Due to the heterogeneous nature of the MDA-MB-231 cell population, the study subsequently investigated the effects of lower doses and combination treatments to optimize therapeutic outcomes.^29^ The inhibition of *CXCR4* prevents breast cancer cells from metastasizing to distant organs such as bones and lungs.^30,31^

Notably, a significant increase in the proportion of cells in the G0/G1 phase was observed after treatment with 100 μM niclosamide (from 45.2% to 61.4%), while the proportions of cells in the S and G2/M phases remained largely unchanged. These findings suggest that niclosamide induces a G1-phase cell cycle arrest, potentially by inhibiting cell cycle progression through mechanisms involving cyclin-dependent kinases (CDKs) or tumor suppressor proteins like p53.^32^ The accumulation of cells in the G0/G1 phase, coupled with subsequent apoptotic death, is often seen in response to unresolved DNA damage or irreparable stress signals.^33^ Han et al. aimed to investigate the therapeutic potential of niclosamide in head and neck squamous cell carcinoma (HNSCC) and to explore its underlying molecular mechanisms. The researchers highlighted the ability of niclosamide to induce cell cycle arrest in the G1 phase and its effects on the Let-7d/CDC34 axis through a series of in vitro and in vivo experiments, including MTT assays, flow cytometry, RT-qPCR, and Western blotting. They demonstrated that niclosamide significantly inhibited HNSCC cell proliferation by inducing G1-phase arrest, leading to suppression of cyclin D1 and activation of p21.^34^

It has been demonstrated that niclosamide modulates apoptotic proteins Bax and Bcl-2 in breast cancer stem cells. Liu et al. demonstrated that niclosamide (0–4 μM, 48 h) combined with cisplatin (0–20 μM) reverses cisplatin resistance in the BT474 breast cancer cell line. They showed that niclosamide exerted its inhibitory effects by inducing apoptosis, downregulating Bcl-2 expression, suppressing the EMT phenotype via decreased levels of N-cadherin and vimentin, and increasing E-cadherin protein levels. Furthermore, inhibition of the STAT3 signaling pathway was confirmed by Western blot analysis. These findings are consistent with our data (Figures 1 and 4).^35^ Vimentin, an intermediate filament protein that supports the epithelial–mesenchymal transition (EMT), contributes to metastasis by enhancing the invasive capability of cancer cells. Our findings indicate that niclosamide suppresses the levels of EMT-related proteins Vimentin and ZEB1. This effect suggests that niclosamide may reduce metastatic potential by inhibiting EMT processes.

The cytokine levels in the table demonstrate the response of cancer stem cells (CSCs) following a 6 h treatment with 100 μM niclosamide. These findings can be attributed to the ability of niclosamide to disrupt multiple oncogenic and proinflammatory pathways. Niclosamide treatment caused a modest increase in IL-8 (from 463.36 ± 42.22 to 508.77 ± 55.08 ng/L) and IL-18 (from 83.80 ± 6.96 to 91.26 ± 1.16 ng/L). IL-8, a chemokine involved in neutrophil recruitment, is known to play a role in angiogenesis and tumor progression by stimulating endothelial cells.^36^ The significant increase in the IL-1β level indicated an activation of inflammatory pathways within the tumor microenvironment. This cytokine is a pivotal mediator of inflammation and has been implicated in enhancing tumor invasiveness by promoting epithelial–mesenchymal transition (EMT) and upregulating matrix metalloproteinase production.^37^ Primary mechanisms of action of niclosamide include the inhibition of Wnt/β-catenin, NF-κB, and STAT3 signaling pathways, which are critical for CSC maintenance, proliferation, and survival. However, in a coculture environment that mimics in vivo conditions, stromal cells such as HUVECs and BM-MSCs can modify CSC responses through paracrine signaling. For example, HUVECs secrete proangiogenic factors like VEGFA and IL-8, while osteoblasts and BM-MSCs contribute to immunomodulation via cytokine release.^38^ Paclitaxel induces apoptosis in cancer cells by inhibiting mitosis and effectively prevents cancer cell proliferation.^39^

In breast cancer, doxorubicin induces downregulation of the Wnt/β-catenin signaling pathway, cell cycle arrest in the G0/G1 phase, an increase in reactive oxygen species levels, and cytotoxic effects.^40^ More recently, it has been described that in breast CSCs, the STAT3 pathway plays a critical role in the conversion of non-CSCs into CSCs through the regulation of the expression of the OCT-4 gene.

CD133 is a commonly used surface marker for identifying cancer stem cells, and CD133+ cells are typically more aggressive and possess tumor-initiating properties. The changes in gene expression observed in coculture systems and between CD133+ and CD133– groups demonstrate the potential of niclosamide to target CSCs. The expression of genes related to metastasis and stemness was found to be suppressed, with this suppression also observed in the more resistant CD133+ cells.^41^ Multidrug resistance (MDR) is one of the most important reasons for chemotherapy failures. ABCG1 and ABCG2 are ATP-binding cassette (ABC) transporter proteins responsible for pumping chemotherapeutic drugs out of cancer cells, thereby contributing to chemotherapy resistance.^42^ ABCG2 reduces the concentration and efficacy of chemotherapeutic agents, such as mitoxantrone and doxorubicin, by scavenging them from the cell. It has been reported that ABCG2 is a stem cell marker with high expression in breast cancer and may be associated with metabolic and signaling pathways such as drug resistance, self-renewal, and invasiveness, and therefore may provide poor prognosis.^43,44^ ABCG2­(+) cell subpopulations in tumors have stem cell-like properties; the central role of ABCG2 in tumor regeneration after chemotherapy has been suggested. In addition, there is evidence that alternative mechanisms (tyrosine kinase inhibitors imatinib and gefitinib) can inhibit ABCG2 both directly and indirectly. This makes these drugs potential candidates for breaking cancer stem cell resistance and targeting these cells.^45^ According to our RT-qPCR results, especially in the CD133+ population, the suppression of the expression of both genes indicates that niclosamide treatment may break the drug resistance in this resistant population. To metastasize, tumor cells must degrade the extracellular matrix using *MMP2* and *MMP9* enzymes, allowing them to migrate to new tissues.^46^ Both *MMP2* and *MMP9* expressions were reduced after niclosamide treatment (*p* < 0.05), which suggests that suppression of these enzymes may reduce the metastatic potential of the tumor. As described in the literature, niclosamide has been shown to inhibit MMP activity, reducing tumor cell motility^47^ and supporting its potential to prevent tumor invasion and metastasis. Although traditionally used as an antiparasitic drug, niclosamide has gained attention as a potential agent in cancer biology, particularly for breast cancer treatment. Niclosamide inhibits the Wnt/β-catenin signaling pathway and suppresses the transcription of Cyclin D1. RT-qPCR results showed that niclosamide significantly decreased the expression of genes such as *CXCR4, MMP2, MMP9, NANOG, CCND1*, these genes play critical roles in cell migration, metastasis, and the maintenance of stem cell-like phenotypic features.^48,49^ Furthermore, the inhibition of stem cell markers (*NANOG* and *OCT4)* in our data indicates that niclosamide reduces the self-renewal capacity of CSCs, thereby preventing their proliferation and tumor formation.^29^ ALDH+ human oral squamous cell carcinoma (OSCC) cells were characterized by upregulated expressions of OCT4, NANOG, and SOX2 and were shown to contribute to the formation of CSCs.^50^ In this study, Wang et al. showed that niclosamide effectively inhibited the activation of the Wnt/β-catenin signaling pathway in ALDH+ CSC-enriched tumorospheres of human OSCC SCC4 and SCC25 cell lines by inhibiting EMT, migration, and colony formation.

Overall, our findings provide further evidence that niclosamide targets key oncogenic pathways in BCSCs, including Wnt/β-catenin, JAK/STAT3, and apoptotic signaling. The observed increase in AXIN2 and decrease in LGR5 suggest that niclosamide disrupts Wnt signaling, thereby impairing stemness. Concurrently, the inhibition of p-STAT3 and modulation of apoptotic markers highlight its potential as a therapeutic agent for eliminating therapy-resistant BCSCs. Future studies should investigate the long-term effects of niclosamide on BCSC populations and explore combination strategies to enhance its efficacy. In this study, we investigated the effects of niclosamide treatment (100 μM, 6 h) on the expression of *AXIN2* and *LGR5* in MDA-MB-231 CD44+/CD24– breast CSCs. Recently, Yi et al. highlighted the critical role of *AXIN2* in the progression of osteosarcoma, particularly through its involvement in the Wnt/SNAIL axis, which is a key driver of EMT that facilitates cancer cell invasion and metastasis.^51^

## Conclusion

Niclosamide demonstrates promising efficacy in targeting CSCs by inhibiting key signaling pathways and downregulating critical genes and protein levels associated with metastasis, stemness, and survival. The reduction in p-STAT3, ZEB1, C-Myc, and Bcl-2 and an increase in the pro-apoptotic marker Bax support its role as a potential therapeutic agent in cancer treatment. Downregulation of the drug resistance markers *ABCG1* and *ABCG2* in CSCs and the CD133+ cells suggests that niclosamide treatment at the selected dose may have reversed the drug resistance. Future research should focus on investigating the long-term effects of niclosamide in vivo, its interactions with other therapies, and its impact on the CSC niche, as well as exploring the molecular mechanisms behind the observed differences in gene expression patterns across different CSC subpopulations.

## Materials and Methods

### Culture and Isolation of CD44+/CD24– Cells from Triple-Negative (ER-/HER2-/PR-) MDA-MB-231 Cells

The triple-negative (ER-/HER2-/PR-) MDA-MB-231 cell line (ATCC) was purchased for this study. The cells were cultured in DMEM/F12 medium (Gibco) supplemented with 10% FBS (Gibco), 2 mM l-glutamine, and 1% penicillin/streptomycin. Plates were maintained under a humidified atmosphere with 5% CO_2_ at 37 °C, and the medium was replaced every 2 days. At 70–80% confluency, cells were washed with PBS, detached using Trypsin-EDTA (Capricorn, 0.5%), and passaged. Subsequently, cells were cultured in DMEM/F12 medium (Gibco) supplemented with 10% FBS (Gibco) at 37 °C and 5% CO_2_ for 3 days.

CD44+/CD24– CSCs were isolated from confluent MDA-MB-231 cells using anti-CD44 and anti-CD24 magnetic beads (Miltenyi Biotech) according to a previously established protocol by our group.^50^ For CSC isolation, the magnetic-activated cell sorting (MACS) method was employed. A cold MACS buffer solution (2–8 °C) containing PBS (pH 7.2), 2 mM EDTA (Sigma), and 0.5% bovine serum albumin (BSA, AppliChem) was prepared. Initially, a CD24-negative cell selection protocol was applied, followed by the isolation of CD44-positive cells. After centrifugation, the cell pellet was resuspended in MACS buffer, and the cell count was determined. The supernatant was removed after centrifugation, and MACS buffer was added to the pellet at a volume of 40 μL per 10^4^ cells. Subsequently, 10 μL of biotin-labeled CD24 (Miltenyi Biotech) per 10^4^ cells was added. The mixture was gently tapped and incubated in the dark at 4 °C for 15 min. After incubation, 1 mL of MACS buffer per 10^4^ cells was added, followed by centrifugation. Once the supernatant was discarded, 80 μL of MACS buffer per 10^4^ cells was added to the pellet. Antibiotin MicroBeads (20 μL per 10^4^ cells) were introduced, and the solution was incubated under the same conditions. An LS column (Miltenyi Biotech) was prepared with 3 mL of buffer, and 500 μL of the cell suspension was filtered through the column. The flow-through containing CD24–/low cells was collected and subjected to CD44+ selection using the same procedure. The expression of surface proteins on CSCs was analyzed by flow cytometry using antihuman CD44-FITC (BioLegend) and anti-human CD24-PE (BioLegend) for phenotypic characterization.

### Assessment of CSC Viability after Targeting Niclosamide

The cells were introduced into a prevascularized 3D bone scaffold using a controlled flow system to ensure even distribution within the structure. Niclosamide was then applied to the system at concentrations ranging from 0 to 100 μM for 6 h to evaluate its effects on cell viability and apoptosis. To assess apoptosis and viability, FITC-Annexin V/7-AAD staining was performed. After treatment, the cells were harvested from the scaffold and washed twice with cold staining buffer to remove any residual medium or debris. The washed cells were then resuspended in Annexin V Binding Buffer at a concentration of 1 × 10^7^ cells/mL. A 100 μL aliquot of the cell suspension was transferred to a 5 mL test tube, and 5 μL of FITC-Annexin V solution along with 5 μL of 7-AAD staining solution were added. The mixture was gently mixed and incubated in the dark at room temperature (25 °C) for 15 min. Following incubation, 400 μL of Annexin V binding buffer was added to the tube, and the samples were analyzed using a flow cytometer (Agilent Novocyte) within 1 h. During flow cytometry analysis, a gating strategy was applied to distinguish and quantify apoptotic cells. Cells positive for Annexin V and negative for 7-AAD were classified as early apoptotic, while those positive for both Annexin V and 7-AAD were classified as late apoptotic.

### Assessment of Protein Levels of CSCs after Targeting Niclosamide

The Annexin-V/7AAD results demonstrated that the IC_50_ value was determined to be 100 μM after 6 h of treatment in breast cancer stem cells (CSCs). Based on this, Western blot was used to evaluate protein levels of the Wnt pathway and apoptosis markers. Protein extracts were prepared using an RIPA buffer (Serva) with protease and phosphatase inhibitors (Thermo Fisher Scientific). Cells were washed with ice-cold PBS, followed by the addition of ice-cold RIPA buffer (100 μL per 1 × 10^7^ cells). Lysates were incubated on ice for 20–30 min, vortexed periodically, and centrifuged at 12 000 × *g* for 10–15 min at 4 °C. The supernatant was collected, and protein concentrations were measured using the BCA assay (Thermo Fisher Scientific). 10% resolving gel and 5% stacking gel were prepared using distilled water, acrylamide/bisacrylamide (Serva), SDS (Sigma), APS (Sigma), and TEMED (Thermo Fisher Scientific). SDS-PAGE was performed, followed by protein transfer to a PVDF membrane and chemiluminescence detection. A total of 30 μg of protein per sample sample was loaded into each well, and electrophoresis was performed to separate the proteins. After electrophoresis, the gel was carefully removed, and proteins were transferred to a PVDF membrane using the Transblot Turbo RTA Transfer Kit (Bio-Rad). The membrane was then blocked with blocking buffer (Nepenthe), followed by incubation with primary antibodies, washing, secondary antibody incubation, further washing, and chemiluminescence detection. Vimentin, C-Myc, EpCAM, ALDH1A1, ZEB1, p-STAT3 (Tyr705), STAT3 (BioLegend), and p-GSK3β (Ser9) (Thermo Fisher) antibodies were used to evaluate protein levels of relevant signaling pathways and EMT markers. Apoptosis was assessed using primary antibodies against Bax and Bcl-2 (BioLegend). HRP activity was visualized using the WesternBright Sirius HRP substrate kit (Advansta), and chemiluminescence imaging was performed using the FluorChem FC3 System (Protein Simple).

### Invasion and Targeting of Cancer Stem Cells in the Three-Dimensional Model

In our previous work, a three-dimensional (3D) model was designed with PLA via a 3D printing method. This scaffold was then filled with a collagen/γ-PGA/Na_2_SiO_3_ hydrogel. It serves as a platform for studying drug release in a fluidic system and was also designed with a cylindrical, vessel-like structure using the electrospinning method incorporating polyurethane (PU). BM-MSCs were added to this material and osteogenically differentiated for 21 days. HUVECs were then added to provide vascularization. In this published article, material and cell characterization was performed.^19^ In the current study, we used a similar 3D design to investigate the invasion response of CSCs within this model after treatment with niclosamide (100 μM). In addition, the invasion of spheroids into the 3D structure filled with the hydrogel material was evaluated and characterized by using a microscope (Olympus IX73).

### Cell Cycle Determination before and after Drug Targeting

Cell cycle analysis was performed before and after niclosamide treatment using flow cytometry to determine the number of cells in the G0, G1, and S phases. After trypsinization, cells were washed, centrifuged, and counted using a hemocytometer by using trypan blue. Following fixation with 96% ethanol (Merck), the tubes were vortexed, and 70 μL of RNase (Sigma, St. Louis, MO, USA) and 100 μL of propidium iodide (Sigma) were added to the cells. The cells were maintained at room temperature in the dark (wrapped in foil) for 20 min and then analyzed using a flow cytometer.

### Evaluation of the Secretion Profile before and after Drug Targeting

The secretion profile of cytokines, chemokines, and growth factors associated with metastasis was assessed in a 3D environment before and after niclosamide treatment. For this, the levels of IL-8, IL-6, IL-1ß, IL-18, and VEGF released into the supernatant after coculture were measured using ELISA according to the manufacturer’s instructions (BT-Lab, China) and detected with a microplate reader (TECAN). Measurements were repeated three times for each sample.

### Evaluation of Gene Expressions before and after Niclosamide Targeting in the 3D Model by RT-qPCR

Prior to and after targeting, cells isolated from enzymatically digested tissue-like structures were subjected to isolation of the resistant group carrying the CD133 marker using anti-CD133 microbeads (Miltenyi) through the MACS method. However, due to the small size of the cell population (1/5 of the total population) and subsequent RT-qPCR analyses, this approach was evaluated in the 3D model. Total cellular RNA was isolated using an RNA isolation kit (Hibrigen) following the manufacturer’s protocol. The RNA concentration was determined by measuring the absorbance at 260 nm using a NanoDrop ND-1000 spectrophotometer (Thermo Fisher Scientific Inc.). RNA quality was assessed based on the A260/A280 ratio. Complementary DNA (cDNA) synthesis was performed by using the High-Capacity cDNA Reverse Transcription Kit (Thermo Fisher). Quantitative PCR was carried out under the following conditions: an initial denaturation step at 95 °C for 10 min, followed by 45 amplification cycles consisting of 95 °C for 10 s, 60 °C for 30 s, and 72 °C for 1 s. A final cooling step at 40 °C for 30 s was included. Each experimental condition was performed in triplicate biological replicates (*n* = 3), with each sample analyzed in triplicate technical replicates. Crossing point (Cp) or threshold cycle (Ct) values for both target and reference genes were determined by using the LightCycler 480 II software. The primers used for gene expression analysis are listed in Table 2.

### Statistical Analyses

Data obtained from the dependent and independent variables in the experiments conducted for this thesis were evaluated for normal distribution using statistical software. Group comparisons were performed using parametric variance analysis tests, specifically one-way ANOVA and Tukey’s test. Each experimental condition was conducted in triplicate (*n* = 3).

## Data Availability

All data needed to evaluate the conclusions in the article are included in the manuscript. Further information and requests for resources or raw data should be directed to, and will be fulfilled by, the lead contact, Betül Çelebi-Saltik: betul.celebi@hacettepe.edu.tr.

## Abbreviations

ABC transporters: ATP-binding cassette (ABC) transporters
ALP: alkaline phosphatase
BM-MSCs: bone marrow mesenchymal stem cells
Col-I: collagen Type-I
CCND1: cyclin D1
CSCs: cancer stem cells
CTCs: circulating tumor cells
ECM: extracellular matrix
ESCs: embryonic stem cells
IPSCs: induced pluripotent stem cells
Micro-Ct: microtomography
MNCs: mononuclear cells
MSCs: mesenchymal stem cells
**Na**_**2**_ **SiO**_**3**_: sodium methasilicate
PBS: phosphate-buffered saline
TICs: tumor-initiating cells
UCB-MSCs: umbilical cord blood mesenchymal stem cells
γ-PGA: poly-gamma-glutamic acid
TNBC: triple-negative breast cancer
